# Clinical studies of detecting COVID-19 from exhaled breath with electronic nose

**DOI:** 10.1038/s41598-022-20534-8

**Published:** 2022-09-26

**Authors:** Andrzej Kwiatkowski, Sebastian Borys, Katarzyna Sikorska, Katarzyna Drozdowska, Janusz M. Smulko

**Affiliations:** 1 Faculty of Electronics, Telecommunications and Informatics, Gdańsk University of Technology, Narutowicza 11/12, 80-233 Gdańsk, Poland; 2 University Center of Maritime and Tropical Medicine, Powstania Styczniowego 9B, 81-519 Gdynia, Poland; 3 Division of Tropical and Parasitic Diseases, Faculty of Health Sciences, Medical University of Gdańsk, Powstania Styczniowego 9B, 81-519 Gdynia, Poland

**Keywords:** Electrical and electronic engineering, Viral infection, Respiratory tract diseases

## Abstract

The COVID-19 pandemic has attracted numerous research studies because of its impact on society and the economy. The pandemic has led to progress in the development of diagnostic methods, utilizing the polymerase chain reaction (PCR) as the gold standard for coronavirus SARS-CoV-2 detection. Numerous tests can be used at home within 15 min or so but of with lower accuracy than PCR. There is still a need for point-of-care tests available for mass daily screening of large crowds in airports, schools, and stadiums. The same problem exists with fast and continuous monitoring of patients during their medical treatment. The rapid methods can use exhaled breath analysis which is non-invasive and delivers the result quite fast. Electronic nose can detect a cocktail of volatile organic com-pounds (VOCs) induced by virus infection and disturbed metabolism in the human body. In our exploratory studies, we present the results of COVID-19 detection in a local hospital by applying the developed electronic setup utilising commercial VOC gas sensors. We consider the technical problems noticed during the reported studies and affecting the detection results. We believe that our studies help to advance the proposed technique to limit the spread of COVID-19 and similar viral infections.

## Introduction

The COVID-19 pandemic created huge demand for proposals for reliable point-of-care testing to reduce its detrimental impact on healthcare systems, and society and business in general. The required features for mass and rapid testing are short time of detection, convenience of sample material collection, and low-cost of use. Polymerase chain reaction (PCR) testing is the gold standard for COVID-19 detection but requires specialist facilities and cannot meet the needs of mass testing in fast pandemic development as required by its omicron version^1^. The PCR test needs a qualified staff to collect the viral material by nasopharyngeal swabs. This process can be easily carried out incorrectly, limiting the number of viral particles necessary for their proper detection.

The proposed rapid at home COVID-19 tests meet these demands to some extent only^2–4^. There is still an urgent need to develop low-cost devices for rapid mass testing. Exhaled breath analysis devices are good candidates because of their non-invasive and less socially inconvenient method, delivering results within a very short time and enabling repeated use^5–7^. There are research studies confirming identification of scents from COVID-19 patients by dogs^8^ and by precise chemical methods (e.g., gas chromatography-ion mobility spectrometry^9^).

Breath samples can be analysed by detecting the SARS-CoV-2 virus or by its indirect indicators as volatile organic compounds (VOCs), exhaled by the infected human body due to metabolic reactions modified by the infection or alteration of the microbial flora of the lungs. The gas cocktail of exhaled VOCs can be detected by an electronic nose device that is less bulky and costly than other breath analysers. Moreover, this method can detect COVID-19 infection during a different development phase than other proposed tests. For example, PCR tests can detect early infection at its early stage, between 3 and 4 days after exposure to the virus.

Successful results of COVID-19 detection by an electronic nose were presented elsewhere by a few independent groups^10–13^. The research applied nanoparticle gas sensors^14,15^, functionalized graphene, or carbon nanotube sensors^16^, and popular metal oxide gas sensors^17^ sensitive to selected VOCs. Some companies have proposed electronic noses that are under clinical studies^18,19^. Moreover, there is experimental evidence of exhaled breath analysis in other viral infections (e.g., influenza^20^, human rhinovirus^21^, and other infections^16^) confirming the production of metabolites leading to exhaled VOCs and their detection by an electronic nose. Recent studies compared specific VOCs present in mechanically ventilated patients with COVID-19 and with non-COVID-19 patients having respiratory acute syndromes^22^. The results distinguished both groups with high accuracy. It is worth mentioning that other human samples (e.g., blood, urine, saliva, or faeces) can be considered for COVID-19 detection, but none of the enumerated samples is as easy to deliver quickly to the analysing device to run the detection test.

There are numerous questions about the mass use of an electronic nose in COVID-19 screening because of the limited repeatability of infection detection and accessible arrays of prototype gas sensors for repeated experiments and practical applications in diverse environmental conditions of breath sample collection. Another issue is unavoidable variations in groups of tested humans. In our exploratory study, we applied a developed electronic nose device utilising commercial gas sensors for VOCs detection and requiring low-energy consumption to operate at elevated temperatures. A detailed description of the electronic nose setup is available elsewhere^23^. The setup can be replicated by others to popularise the proposed method and to introduce necessary modifications to enhance the detection results and hasten possible mass application.

The clinical studies were run within a group of patients in the north part of Poland. The breath samples were collected and analysed in the University Center of Maritime and Tropical Medicine, Gdynia, Poland by the developed electronic nose in the Gdańsk University of Technology. The research teams gained experience in the electronic nose technique by participating in the EU project TROPSENSE a few years ago^24,25^.

## Methods of experimental studies

### Investigated cohort

Our experimental studies were run in a hospital ward dedicated to the treatment of COVID-19 infected patients during the third wave of the pandemic in Poland, in the period between March and July 2021. The SARS-CoV-2 Alpha and Delta variants dominated within this period in Poland. The breath samples were collected in the clinic rooms within a group of the hospitalised patients and a control group. We investigated 56 breath samples (33 patients with severe course of COVID-19 disease, 17 breath samples collected within the healthy control group, and 6 samples of ambient air). A detailed description of the investigated group is presented in Table S1, in the supplementary information. The number of scrutinised samples related to the contemporary pandemic situation in the north of Poland and patients admitted to the hospital ward. We are conscious that a more abundant cohort should be applied to determine a significance level of the proposed COVID-19 infection testing when limited only to a few percentages. We investigated patients of different age, gender, and accompanying diseases. The anonymised patient data list their accompanying diseases, medications taken, and blood concentration of C-reactive protein (CRP), associated with inflammation in the human body.

### Breath sample collection

**Figure 1:**
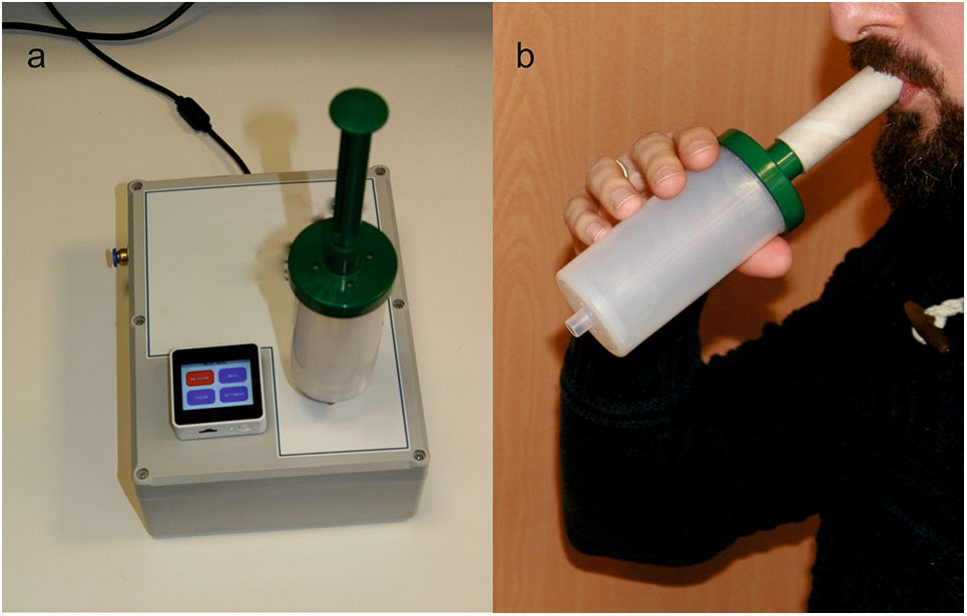
(**a**) Developed electronic nose analyzing the sample of exhaled breath by using the end-tidal part the final wave, and (**b**) illustration of collecting the breath sample with the BioVOC™.

All samples were collected in the morning before eating or drinking, except for fresh water, or brushing their teeth to reduce the detrimental impact of food or drink smells. The samples were collected from the patients and healthy volunteers breathing the same atmosphere of hospital ward rooms. The end-tidal part of the final wave of exhaled breath was collected by a BioVOC^TM^ breath sampler (about 130 mL), made of Teflon, and comprised of a protective one-way flow valve, see Figure 1. The BioVOC^TM^ breath sampler was cleansed after each use by dismantling and inserting into a solution of 20 mL of disinfectant (Milton, France) dissolved in 1 L of distilled water. The breath sampler was left to dry naturally after 15 min of staying in the disinfectant solution. The collected breath sample was introduced into the gas chamber of the electronic nose (Figure 1a) by attaching the BioVOC^TM^ to the inlet of the gas chamber under low pressure and automatically opening an electrical valve.

The applied procedure of exhaled breath sampling prevented any accidental virus contamination between the examined patients due to the high contagiousness of the SARS-CoV-2 virus. It can be easily simplified to accelerate this process in the near future. A good technical solution is to blow directly into the inlet of the gas chamber with the sensors and use a micro-phone to detect the beginning of the end-tidal part of the final wave, followed by its introduction into the gas chamber^26,27^.

### Electronic nose setup

**Figure 2:**
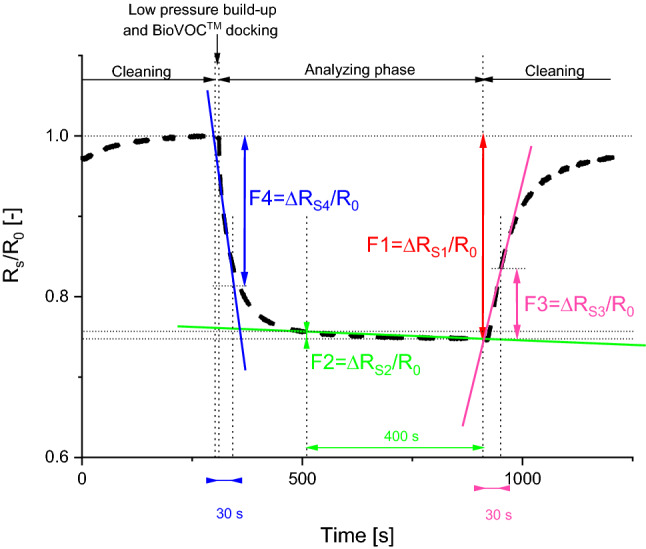
Illustration of the recorded relative changes of gas sensor DC resistance *R*_S_ during breath sample analysis, related to its initial resistance *R*_0_ after sensor cleaning. The four parameters of DC resistance changes F1, F2, F3, and F4 were evaluated to present the sensor’s response to the introduced breath sample.

Our setup applied a prolonged sensor cleaning process by sensor insufflation using ambient air. All highlighted stages of electronic nose operations are presented in Figure 2. We start with cleaning the gas sensors via a continuous flow of ambient air through the gas chamber (between 2 and 3.2 L/min). It purged the gas chamber with sensors by ambient air many times and can be shortened to reduce the time of measurement procedure in an updated electronic nose version. Next, the electric pump creates a low pressure (about 350 hPa) region in the gas chamber within 30 s and sucks the breath sample from the BioVOC^TM^ into the gas chamber. The electric valve closes the gas chamber, and we observe the gas sensors’ responses to the introduced breath sample.

We applied a set of four parameters, representing relative changes of the sensor DC resistance *R*_S_, observed in the selected time intervals, as presented in Figure 2: F1 = Δ*R*_S1_/*R*_0_ (maximum relative change of DC resistance within analysing phase); F2 = Δ*R*_S2_/*R*_0_ (relative change of DC resistance within the final part of the analysing phase—the last 400 samples); F3 = Δ*R*_S3_/*R*_0_ (a tangent slope of DC resistance relative change at the beginning of the cleaning phase—evaluated within the period of the first 30 samples of the cleaning phase which follows the analysing phase); F4 = Δ*R*_S4_/*R*_0_ (a tangent slope of DC resistance relative change at the beginning of the analysing phase—evaluated within the period of the first 30 samples). These parameters depend on the difference in the recorded DC resistances and reduce an impact of unavoidable drift in time of the applied resistive gas sensors on detection results. The proposed parameters can be automatically evaluated from the recorded time series of the sensors’ DC resistances. Moreover, the proposed parametrisation reduced the number of the analysed data points by giving the averaged parameters, which is more robust against the noise and interferences that are naturally present in the recorded time series.

The electronic nose device was tested in a laboratory to determine the repeatability of the recorded time series by analysing consecutively collected exhaled breath samples from the same volunteer. We observed a change of up to 15% in the time series of the recorded DC resistances at the same points in the measurement procedure (Analysing phase—Figure 2). We applied MATLAB scripts, prepared for the release R2021b, and used slope evaluation functions—see the supplementary information. The selected parameters reduced the detrimental effect of the gas sensors’ slow drifts in time, induced by ageing or gradually varying fluctuations in the composition of the ambient atmosphere^28^. This disturbing effect was considered in numerous studies and is one of the most significant and reducing possibilities for measuring low concentrations of VOCs and any medical applications of high efficiency in practice^29^. We underline that in a case of COVID-19 patients there are experimental studies confirming that the environmental pollutants, present in the ambient air (due to, e.g. air conditioning systems) have a harmful impact on the effectiveness of medical treatment and therefore can also provide useful information^30,31^.

We applied a set of commercial gas sensors designed to monitor selected VOCs (supplementary information, Table S3). The sensors were developed within the last few years by the leading companies in the gas sensing industry. Integrated circuit technology was applied for their construction to optimise their energy consumption, which is necessary for operating at elevated temperatures, and to ensure repeatability of their parameters. The sensors are dedicated to portable applications, which is an emerging area of gas sensing applications.

The same gas sensors were applied in electronic noses designed by other research groups and aimed at similar medical or environments applications^23,26,32^. The selected sensors focus on various VOCs present in indoor environmental or specifically dedicated to the VOCs in exhaled breath. The electronic nose monitored the environmental conditions (temperature, humidity, and pressure) during collection of the breath samples to check their repeatability during the studies. The recorded time series for exemplary COVID-19 and healthy patients are presented in supplementary information, in Figs. S1 and S2, respectively. We observed remarkable differences in the shapes of the recorded DC resistances for the COVID-19-diagnosed patients (Figure S1a) and the healthy patients (Figure S2a) from the control group. The differences were visible for the applied gas sensors. The exception was the BME680 gas sensor, which responded too slowly to be used in practice for the analysed breath samples, and therefore these data were excluded from further consideration. We observed some differences in environmental conditions between the patients, especially for humidity (Figures S1b, S2b) in the initial part of the analysing phase. The differences did not exceed 15% and were much less intense than identified for the applied gas sensors.

The electronic nose recorded voltages across the gas sensors operating in serial connection with resistors as independent voltage dividers, supplied by precise voltage references of 2.5 V (REF192; Analog Devices). Voltages were sampled by 16-bit low-power analogue-to-digital converters (ADS1115; Texas Instruments). The developed electronic nose comprised of more gas sensors than the applied for COVID-19 detection algorithm. We limited the built-in gas sensors to the sensors securing a stable response within the assumed time of the analysing phase. We underline that some commercial gas sensors can be potentially used for COVID-19 but require preconcentrated breath samples to accelerate their response time or their composition should be modified (e.g., by introducing noble metals or UV irradiation to enhance the gas sensitivity of the applied metal oxide sensing layers)^33,34^. Further improvement can be reached by applying nanoparticle technology of enhanced sensitivity or two-dimensional materials, which exhibit a high ratio of the active area to its volume and reaching the detection level of a single molecule^35^. A great improvement in the gas selectivity of resistance gas sensors was reported for organically functionalised golden nanoparticle prototype structures that are not available commercially^36,37^.

The applied setup secured stable conditions for measuring the gas sensors’ response in the analysing phase (Figure 2). The proceeding and following cleaning phases displayed visible fluctuations in the sensors’ DC resistances, induced by non-stabilised laboratory airflow through the gas chamber. We noticed sharp changes in gas sensors response before closing the electrical valve at the beginning of the analysing phase. This is an effect of introducing the breath sample into the gas chamber, which can be controlled more accurately by a few technical modifications. Firstly, we can control the pump to manufacture a more stable/repeatable low-pressure zone. Secondly, more precise adjustment between the inlet into the gas chamber and the BioVOC^TM^ sample secures rerun process of breath sample transmission. We can also introduce the breath sample without using the BioVOC^TM^ but utilising a pump electronically controlled to identify the tidal-wave breath final phase that is introduced into the gas chamber. Further improvement can be reached by applying preconcentration of the analysed VOCs, as presented elsewhere, but requiring a bulkier and more energy-consuming setup^38^. Some of these underlined detrimental effects can be corrected with simple changes, for more accurate control of the flow of the breath sample.

### Data analysis

The collected time series were automatically parameterised (Fig. 2) to reduce the sets of analysed numbers. We estimated the selected slopes at defined time intervals by using Matlab scripts. The detailed data for the investigated cohort were included into the supplementary information (Table S1). Each patient was anonymised, described by a unique number and health status (COVID-19-infected or healthy patient). Each breath sample was described by four parameters delivered from five gas sensors (Fig. S1a), and three recorded environmental quantities (Fig. S1b). The applied Matlab scripts, used for sensor response parametrisation, are available in the supplementary information (Section 3. Matlab scripts).

**Figure 3:**
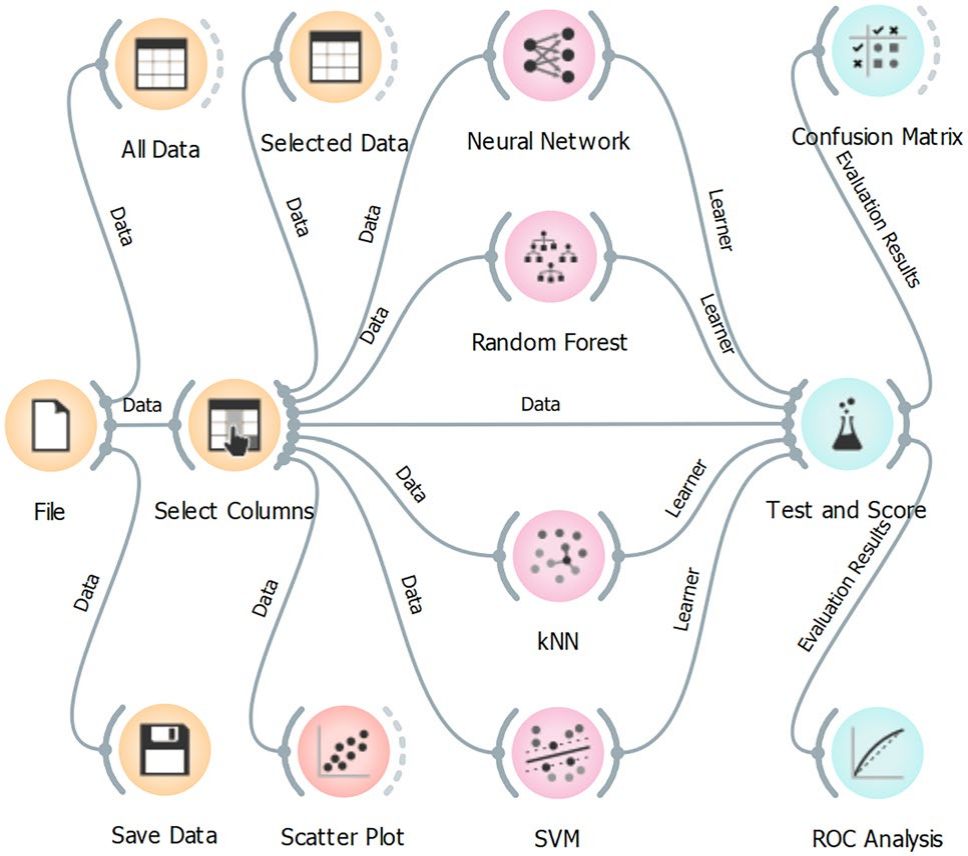
The graph structure of the Orange software implementing the selected detection algorithms (Neural Network, Random Forest Data, kNN, SVM) and compared by using stratified cross-validation method (*Test and Score*) by 5-folds. The results are presented by receiver operating characteristic curve (*ROC Analysis*) and an error matrix (*Confusion Matrix*).

Next, a few selected algorithms were applied to determine the efficiency of COVID-19 detection. There is abundant literature presenting various methods of data analysis in olfactory applications^39–42^. We applied the algorithms available in the Orange software^43^. This is a hierarchically-organised piece of software that implement data-mining algorithms by front-end visual programming. Data processing was run by implementing a graph structure, determining all steps of the data processing (Figure 3).

We applied four algorithms to perform the detection task: (i) a multi-layer perceptron algorithm (*Neural Network* widget)^44^, (ii) random forest^45^, (iii) k-nearest neighbours algorithm (kNN)^46^, and (iv) support vector machine (SVM) algorithm^47^ which implements the LIBSVM library to execute the SVM algorithm^48^.

Multi-layer perceptron is a supervised learning algorithm that approximates the input data into the given output by non-linear function in general. The default parameters were applied for this algorithm (neurons in the hidden layers: 100; activation function for the hidden layer: rectified linear unit function—ReLu; maximum number of iterations: 200). Random forest algorithm is a prediction learning method that is also used for classification. It builds a set of decision trees by considering the analysed data. The class selected by most of the trees is the decision result. The algorithm requires a selection of the applied number of trees (the default number of 10 trees was applied). The kNN algorithm predicts the detection results by considering the nearest training instances (e.g., by using a weighted average of the k nearest neighbours) to reduce the amount of the considered data. We applied the default number of 5 nearest neighbours. The SVM algorithm classifies the data by applying non-linear functions to determine a hyperplane of the maximised margin between the classified groups (e.g., COVID-19-infected and healthy patients). We applied radial basis function (RBF) and default parameters to determine the time of the necessary computations. The briefly mentioned and applied algorithms are commonly used for detection goals in electronic nose data analysis, including medical applications^39,49^.

The results of the selected detection algorithms were tested to evaluate their classification accuracy by stratified cross-validation technique^50^. We applied the *Test and Score* widget in the Orange software and split the data into five folds to run the method that randomly splits the data into 5 equal-sized sets for testing the model (one subsample) and training data (four subsamples) in a way that the proportions between decision classes (33 COVID-19-infected and 17 healthy patients) were preserved in all sets. All algorithms were tested and the results present receiver operating characteristic (ROC) curves (*ROC Analysis* widget—Figure 3) and confusion matrixes (*Confusion Matrix* widget—Figure 3). The ROC curve plots the true positive (TP—correctly identified COVID-19 infected patients) versus false positive (FP—healthy patients and ambient air incorrectly identified as COVID-19 infected) rates. The ROC curve determines the dependence between sensitivity (TP) and FP (1—specificity). The performance of the applied algorithms can also be evaluated by the data delivered by the *Test and Score* widget. Selected data are included in the supplementary information (Section 4. Detection efficiency).

### Ethical approval and informed consent

All subjects gave their informed consent for inclusion before they participated in the study. The study protocol and all experimental procedures were approved by the Independent Bioethical Commission for Science Research at the Medical University of Gdańsk, Poland (ethical approval code: NKBBN/501/2020). All methods were performed in accordance with the relevant guidelines and regulations.

## Results

The collected data were pre-analysed by scrutinising the evaluated parameters of the gas sensors (Figure 2). We excluded the parameter F4 because of its more extensive variability concerning the other considered parameters within the applied gas sensors. Its variability also results from construction of the electronic nose, as suggested above, and therefore should be excluded from further consideration. We applied the F1, F2, and F3 parameters which characterise the response of five gas sensors: GM-402B, MiCS-6814 (CO, NO_2_, NH_3_), TGS8100 and an environmental humidity sensor. The response of the gas sensors to the exhaled breath between the COVID-19-infected and healthy groups depended firmly on the sensor and considered parameter.

**Figure 4:**
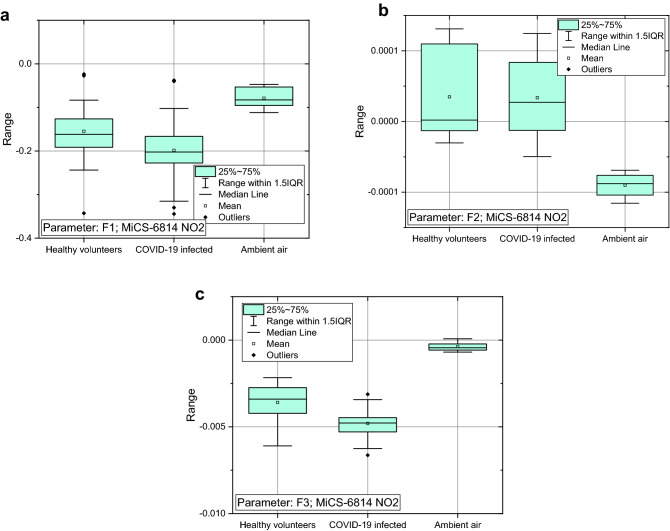
Difference in response of the MiCS-6814 (NO_2_) gas sensor between the groups of COVID-19-infected patients (33), healthy volunteers (17) and ambient air (6) for three selected parameters of major difference: (**a**) F1, (**b**) F2, and (**c**) F3. IQR –interquartile range, presenting the statistical dispersion of the considered data.

The response of the MiCS-6814 (NO_2_) sensor to the three groups of the investigated cohort was compared statistically (Figure 4). It is evident that the response of this sensor for COVID-19-infected patients sticks out statistically from the response of the two other groups. The parameters F1, and F3 for the groups of healthy volunteers and ambient air stand out in the same direction from the parameters for the COVID-19-infected patients, but of different intensity (e.g., compare parameters: F1—Figure 4a, and F3—Figure 4c). The result confirms that there is a distinctive response of the gas sensors between these groups. The results observed for the sensors TGS8100 (Fig. S3 in supplementary information), MiCS-6814 (CO—Fig. S4 in supplementary information, and NO_2_—Figure 4) suggest that the most distinctive are the parameters F1 and F3, but the more in-depth analysis is required for a larger cohort, including their impact on the applied detection algorithm.

**Figure 5:**
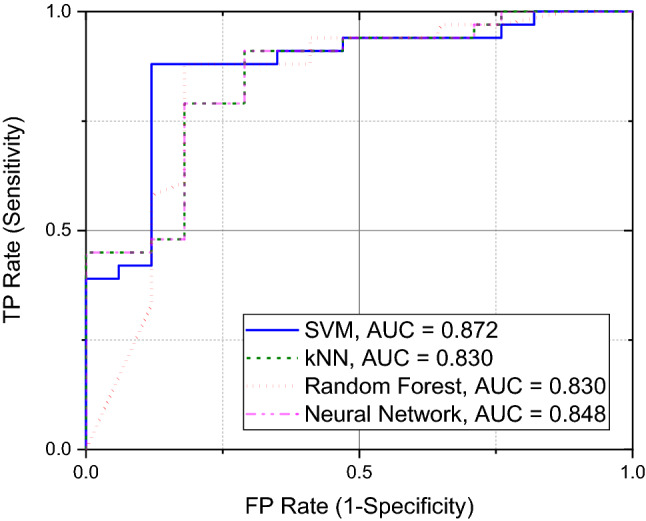
ROC curves observed for the examined cohort of 50 samples (33 COVID-19-infected patients, 17 healthy volunteers). Four selected detection algorithms were considered: SVM, kNN, Random Forest, Neural Network. AUC—Area Under the Curve.

We explored the classification algorithms within the two groups (COVID-19-infected patients and control group) to evaluate the detection results and the impact of environmental conditions and age. The ROC curves were determined for the cohort of 50 (33 COVID-19-infected patients, 17 healthy volunteers—Figure 5). It is important to draw attention to the fact that ambient air (filtration and circulation) can bias the detection results, but can also have a serious impact on the efficiency of medical treatment, as reported elsewhere^30,31^. Therefore, it is important to consider its impact on COVID-19 detection or even continuous monitoring in hospital wards by an electronic nose to enhance the efficiency of medical treatment.

**Figure 6:**
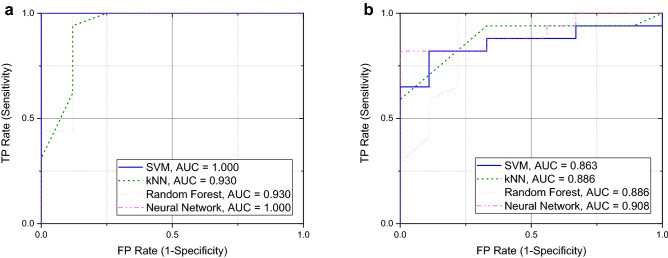
ROC curves observed for the examined cohort of (**a**) 24 samples (16 COVID-19-infected patients over 55 years old, 8 healthy volunteers over 45 years old), and (**b**) 26 samples (17 COVID-19-infected patients under 55 years old, 9 healthy volunteers under 45 years old). Four selected detection algorithms were considered: SVM, kNN, Random Forest, Neural Network. AUC—Area Under the Curve.

The developed discriminating models presented excellent training and blind divergence results for all considered algorithms. For example, the area under the ROC was above 0.83 for these algorithms, and for both cohorts (Figure 6a,b). Discrimination between COVID-19-infected and healthy patients only resulted in 84% classification accuracy for the Neural Network and Random Forest algorithms. Worse results of 70% classification accuracy were observed for the kNN algorithm. Exclusion of the humidity sensor data from analysis resulted in a drop of classification accuracy to 66% only for the kNN algorithm. The other algorithms secured better classification accuracy between 70% and 74%. It means that aggravated COVID-19 infection modifies the exhaled VOCs but also the humidity in breath samples to some extent. The detailed results for all algorithms and the considered cohorts are presented in the supplementary information (Tables S4–S6). The detection outcome suggests quite similar efficiency of the applied algorithms for the considered and limited cohort, without the set of ambient air samples.

COVID-19 infection is more severe for the health of elderly patients and therefore we can expect statistically more vivid changes in their exhaled breath than for younger patients. We divided the cohort into two subsets using as a threshold the median age of the COVID-19-infected (55 years) and healthy volunteers (45 years) separately. The evaluated ROC curves for the subset of older (equal to or over the median age—Figure 6a) and younger (under the median age—Figure 6b) confirmed better detection results for the older group, where the classification accuracy reached over 91% for all algorithms (Table S5). Detection accuracy dropped to 80% for the group of younger patients in the worst-case Neural Network algorithm (Table S6). These results endorse the proposed methodology of electronic nose use that is statistically more effective for exposing the most troubled elderly patients.

## Discussion

The detection results suggest the significant potential of the proposed method by using commercial gas sensors. Similar results were reported by other groups and resistive sensors elsewhere^10–13^. We are convinced that our approach can be advanced and applied in practice even if the presented results were observed in the unique environmental conditions of hospital wards. The limited number of observations presented in our studies, heterogeneity of the investigated groups (healthy volunteers versus COVID-19 infected), several possible confounding effects (e.g., medical treatment, accompanying diseases) require further in-depth studies before an application for mass COVID-19 pre-screening testing. Outdoor use of exhaled breath testing is a challenge that will certainly limit detection results. A few companies have proposed similar electronic nose units recently^18,19^. Unfortunately, no statistics present their detection results in practice for the time being. We underline that our results exposed a much better detection rate for the elderly group. Moreover, we noticed that there was a difference in the humidity of the analysed breath samples. COVID-19-infected patients displayed higher humidity than healthy volunteers on average. In our exploratory studies, we focussed on possible amendments that could improve testing accuracy and resistance to fluctuating environmental factors. We identified the most reliable parameters of gas sensors that can be applied during a test with a substantially shortened time. Further enhancement requires sensors of better selectivity and sensitivity, as underlined elsewhere^10^. The concentrations of separate VOCs markers of COVID-19 infection are in the tens of ppb level, as reported elsewhere^22^. Still, the cocktail of numerous VOCs in exhaled breath has sufficient impact on the applied commercial gas sensors to detect COVID-19 infected patients.

There are still a few open questions before any application in COVID-19 testing. We do not know how the unavoidable time drift of resistive gas sensors affects the detection rate by the electronic nose and the need for its repeated calibration during intensive operation. A more efficient and fast procedure for gas sensor cleaning should be introduced (e.g., by applying UV-irradiation or pulse heating). The next problem is detection efficiency in open air conditions and in the presence of other pulmonary diseases (colds, seasonal flu, etc.). An open question is the application of pre-concentrator techniques to increase the VOC concentrations and reduce the demands placed on the gas sensors. This technique will increase costs and make the electronic nose more bulky. We believe that the proposed methodology is a step forward to introduce mass COVID-19 screening and monitoring its medical treatment efficiency. This method should be faster and low-cost, but will definitely not be as accurate as the gold standard PCR test.

## Supplementary Information

Supplementary Information.

## Data Availability

The detailed data that support the findings of our experimental study are available from the corresponding author upon reasonable request.
