# Supplementary material for: Clinical studies of detecting COVID-19 from exhaled breath with electronic nose
Source: Sci Rep. 2022 Sep 26;12:15990. doi: 10.1038/s41598-022-20534-8 (PMC9512806; doi:10.1038/s41598-022-20534-8)
Supplement: Supplementary file 1 — Supplementary Information. [file 41598_2022_20534_MOESM1_ESM.pdf]

Supplementary information

Clinical Studies of Detecting COVID-19 from Exhaled Breath with Electronic Nose

Andrzej Kwiatkowski[1], Sebastian Borys[2], Katarzyna Sikorska[2,3], Katarzyna Drozdowska[1], and Janusz M. Smulko[1,*]

[1]Faculty of Electronics, Telecommunications and Informatics, Gdańsk University of Technology, Narutowicza 11/12, 80-233 Gdańsk, Poland
[2]University Center of Maritime and Tropical Medicine, Powstania Styczniowego 9B, 81-519 Gdynia, Poland
[3]Division of Tropical and Parasitic Diseases, Faculty of Health Science, Medical University of Gdansk, Powstania Styczniowego 9B, 81-519 Gdynia, Poland
[*]janusz.smulko@pg.edu.pl

1. Questionnaire

The volunteers completed an anonymised questionnaire concerning their health, smoking habit and pharmacological treatment. The collection date and time were recorded. The detailed data for the investigated cohort of COVID-19-infected and healthy patients are presented in Table S1. The breath samples were collected in the hospital wards of the University Center of Maritime and Tropical Medicine, Gdynia, Poland, as indicated in the table.

Table S1. Anonymised questionnaire data of the cohort of 56 investigated samples (33 COVID-19-infected patients, 17 healthy volunteers, 6 ambient air).

No.	Collection Date and Time	Place (Room No.)	Fever days	Days since diagnosis COVID-19	Other Diseases	Age range (years)	Gender	Smoker (Yes/No)	CRP concentra tion [mg/l]	Days since CRP test	Medication
1	09.04.21, 08:15	123	9	8	Arterial hypertension, hypercholesterol emia, obesity	40–49	Male	No	167	1	Remdesiwir, Ceftriaksone, Dexamethasone, Oxygen
2	14.04.21, 08:15	129	9	13	Prostate hypertrophy,	>60	Male	No	155	0	Ceftriaksone, Enoksaparin
3	15.04.21, 08:30	126	9	8	arterial hypertension	>60	Female	No	227	0	Ceftriaksone, Enoksaparin, Oxygen

4	15.04.21, 08:45	129	12	3	-	>60	Male	No	154	1	Ceftriaksone, Enoksaparin, Oxygen
5	16.04.21, 08:15	105	0	7	Obesity	40–49	Male	No	77	0	Remdesiwir, Ceftriaksone, Dexamethasone, Oxygen, Enoksaparin
6	16.04.21, 08:30	131	7	4	Arterial hypertension, gout, prostate hypertrophy	50–59	Male	No	55	0	Dexamethasone, Enoksaparin, Oxygen
7	16.04.21, 08:45	129	11	9	-	>60	Male	No	70	0	Remdesiwir, Ceftriaksone, Dexamethasone, Oxygen
8	16.04.21, 08:55	128	4	2	Arterial hypertension	>60	Male	No	18	0	Dexamethasone, Enoksaparin, Ceftriaksone, Oxygen
9	19.04.21, 08:15	104	5	5	Arterial hypertension, hypothyroidism, schizophrenia	>60	Female	No	41	0	Dexamethasone, Ceftriaksone, Klozapol, Oxygen
10	21.04.21, 08:10	129	15	13	-	>60	Male	No	9	0	Remdesiwir, Dexamethasone, Ceftriaksone, Oxygen
11	21.04.21, 08:15	129	10	2	Adrenal insufficiency	>60	Male	No	367	2	Ceftriaksone, Remdesiwir, Dexamethasone, Oxygen
12	21.04.21, 08:20	128	7	6	Osteoarthritis of the hip joints	50–59	Male	No	18	0	Enoksaparin, N-Acetylcysteine, Oxygen
13	21.04.21, 08:25	128	10	8	Arterial hypertension, liver damage	>60	Male	No	3	0	Ceftriaksone, Dexamethasone, Oxygen
14	23.04.21, 08:10	128	10	9	-	50–59	Male	No	18	2	Enoksaparin, Oxygen

15	23.04.21, 08:15	128	10	10	Arterial hypertension	30–39	Male	No	57	2	Enoksaparin, Favipiravir, Oxygen
16	28.04.21, 08:10	128	9	5	Arterial hypertension	50–59	Male	No	283	0	Remdesiwir, Dexamethasone, Ceftriaksone, Oxygen
17	29.04.21, 08:10	129	8	8	Atrial fibrillation, heart failure, hypothyroidism	>60	Male	No	15	0	Ceftriaksone, Rivaroxaban, Levothyroxine, Oxygen
18	29.04.21, 08:15	128	15	14	History of hip arthroplasty	50–59	Male	No	66	0	Enoksaparin, N-Acetylcysteine, Oxygen
19	29.04.21, 08:20	128	15	14	Arterial hypertension	30–39	Male	No	102	1	Favipiravir, Ceftriaksone, Dexamethasone, Oxygen
20	05.05.21, 08:05	130	14	13	Atrial fibrillation, hypothyroidism	>60	Female	No	10	2	Dabigatran, Levothyroxine, Indapamide
21	05.05.21, 08:10	104	11	8	-	50–59	Female	No	7	0	Remdesiwir, Ceftriaksone, Dexamethasone, Oxygen
22	05.05.21, 08:15	128	11	7	Bronchial asthma	50–59	Female	No	73	0	Remdesiwir, Ceftriaksone, Deksametazon, Enoksaparin, Oxygen
23	06.05.21, 08:10	130	10	7	Rheumatoid arthritis, dyslipidemia	50–59	Female	No	13	0	Remdesiwir, Dexamethasone, Ceftriaksone
24	06.05.21, 08:15	128	0	7	Emphysema, gout, fatty liver, atrial fibrillation	>60	Male	No	73	0	Ceftriaksone, Rivaroxaban, Allopurinol
25	12.05.21, 08:10	103	14	8	Rheumatoid arthritis, arterial hypertension, renal failure, bronchial asthma	>60	Female	No	80	1	Ceftriaksone, Dexamethasone, Enoksaparin

26	12.05.21, 08:15	128	0	20	Arterial hypertension	50–59	Male	No	8	1	Enoksaparin, Codeine, Oxygen
27	14.05.21, 08:05	128	0	5	Bronchial asthma, obesity	18–29	Male	No	1	0	Methylprednisolon, Budesonide, Ipratropium bromide, Oxygen
28	14.05.21, 08:10	128	14	15	Bronchial asthma, obesity, arterial hypertension	50–59	Male	No	10	2	Remdesiwir, Ceftriaksone, Dexamethasone, Enoksaparin, Oxygen
29	25.05.21, 08:10	104	15	12	Arterial hypertension, hepatitis B virus	40–49	Male	No	56	1	Remdesiwir, Ceftriaksone, Enoksaparin, Oxygen
30	27.05.21, 08:05	128	12	8	Hypothyroidism, obesity	50–59	Female	No	25	0	Remdesiwir, Ceftriaksone, Enoksaparin, Oxygen
31	02.06.21, 08:05	123	23	21	Follicular lymphoma, polyneuropathy	40–49	Male	No	79	2	Remdesiwir, Ceftriaksone, Enoksaparin, Oxygen
32	02.06.21, 08:10	123	Ambient air	Ambient air	-	Ambient air	Ambient air	Ambient air	Ambient air	Ambient air	-
33	02.06.21, 08:15	123	Ambient air	Ambient air	-	Ambient air	Ambient air	Ambient air	Ambient air	Ambient air	-
34	04.06.21, 08:15	134	Healthy	Healthy	-	50-59	Female	Nie	Healthy	Healthy	-
35	04.06.21, 08:20	129	Ambient air	Ambient air	-	Ambient air	Ambient air	Ambient air	Ambient air	Ambient air	-
36	04.06.21, 08:25	129	Ambient air	Ambient air	-	Ambient air	Ambient air	Ambient air	Ambient air	Ambient air	-
37	07.06.21, 08:35	134	Healthy	Healthy	-	40–49	Male	No	Healthy	Healthy	-
38	07.06.21, 08:40	128	Ambient air	Ambient air	-	Ambient air	Ambient air	Ambient air	Ambient air	Ambient air	-
39	08.06.21, 07:00	134	Healthy	Healthy	-	30–39	Male	No	Healthy	Healthy	-
40	08.06.21, 08:30	134	Healthy	Healthy	-	18–29	Male	No	Healthy	Healthy	-

41	08.06.21, 08:35	134	Healthy	Healthy	-	50–59	Female	No	Healthy	Healthy	-
42	08.06.21, 08:40	129	Ambient air	Ambient air	-	Ambient air	Ambient air	Ambient air	Ambient air	Ambient air	-
43	10.06.21, 08:05	104	10	7	-	50–59	Male	No	185	2	Ceftriaksone, Dexamethasone, Oxygen
44	15.06.21, 07:40	104	0	4	Kidney transplant in 2006, arterial hypertension, thyroid nodules	>60	Female	No	38	1	Methylprednisolone, tacrolimus, mycophenolate mofetil, meropenem
45	22.06.21, 07:35	134	Healthy	Healthy	-	18–29	Male	No	Healthy	Healthy	-
46	29.06.21, 06:00	134	Healthy	Healthy	-	50–59	Female	No	Healthy	Healthy	-
47	29.06.21, 06:10	134	Healthy	Healthy	-	50–59	Female	Yes	Healthy	Healthy	-
48	29.06.21, 07:00	134	Healthy	Healthy	-	18–29	Female	Yes	Healthy	Healthy	-
49	06.07.21, 07:00	134	Healthy	Healthy	-	50–59	Female	No	Healthy	Healthy	-
50	06.07.21, 07:10	134	Healthy	Healthy	Obesity	50–59	Female	No	Healthy	Healthy	-
51	06.07.21, 07:15	134	Healthy	Healthy	Hypothyroidism	50–59	Female	Yes	Healthy	Healthy	Levothyroxine
52	06.07.21, 07:30	134	Healthy	Healthy	-	18–29	Female	Yes	Healthy	Healthy	-
53	13.07.21, 06:00	134	Healthy	Healthy	-	40–49	Male	No	Healthy	Healthy	-
54	13.07.21, 06:10	134	Healthy	Healthy	Obesity	40–49	Female	No	Healthy	Healthy	-
55	13.07.21, 06:15	134	Healthy	Healthy	-	40–49	Female	No	Healthy	Healthy	-
56	13.07.21, 06:45	134	Healthy	Healthy	-	18–29	Female	Yes	Healthy	Healthy	-

Table S2. A statistical summary of the selected groups: 33 COVID-19-infected patients, 17 healthy volunteers.

Group	Mean number of fever days	Mean number of days since diagnosis COVID-19	Other Diseases	Age range (years)	Gender	Smoker (Yes/No)	CRP concentration [mg/l]	Days since CRP test	Medication
COVID-19-infected patients	9.4	8.8	Yes – 27<br>No – 6	18–29: 1<br>30–39: 2<br>40–49: 4<br>50–59: 12<br>>60: 14	Male: 24<br>Female: 9	Yes – 27<br>No – 6	77.6	0.64	Yes – 33<br>No – 0
Healthy volunteers	-	-	Yes – 3<br>No – 14	18–29: 5<br>30–39: 1<br>40–49: 4<br>50–59: 7<br>>60: 0	Male: 5<br>Female: 12	Yes – 27<br>No – 6	155	-	Yes – 1<br>No – 16

2. Gas sensors

Commercial gas sensors designed to monitor selected volatile organic compounds were used for the experiment. The detailed list of the sensors is presented in Table S3. The recorded time series of DC resistances and environmental conditions for exemplary COVID-19 and healthy patients are presented in Figure S1 and Figure S2, respectively.

Table S3. List of the metal oxide gas sensors dedicated to monitor volatile organic compounds and environmental conditions.

Gas sensor	Detected gases/environmental conditions
GM-402B (Winsen)	C_3H_8, CH_4 (combustible gases)
MiCS-6814 (SGX Sensortech)	Three independent sensing elements: CO, NO_2, NH_3 (agriculture/industrial odors)
TGS8100 (Figaro)	H_2, CO, CH_4, H_2, C_2H_5OH, $HC(CH_3)_3$ (air contaminants)
BME680 (Bosch Sensortec)	CO, C_2H_6, C_2H_5OH, C_5H_8, C_3H_6O (indoor air quality and exhaled breath VOCs; temperature *T*, humidity *H*, pressure *P*)

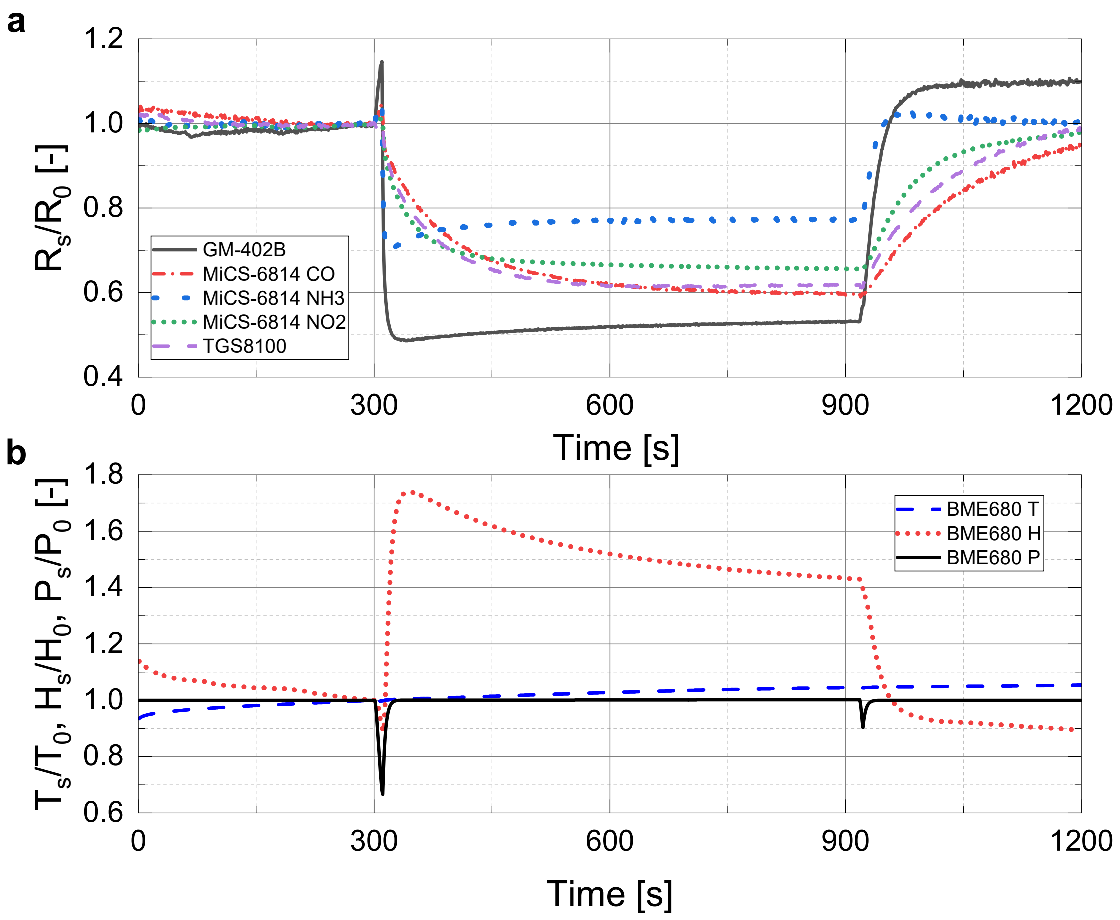

Figure S1. Exemplary time series of (a) DC sensor resistance R_S relative to its initial value R_0 for the applied gas sensor and, (b) environmental conditions (temperature T, humidity H, and pressure P) related to their initial values (T_0, H_0, and P_0 respectively) for a COVID-19-diagnosed patient. The legend shows the applied sensor according to the names in Table S3 representing the detected gases or environmental condition.

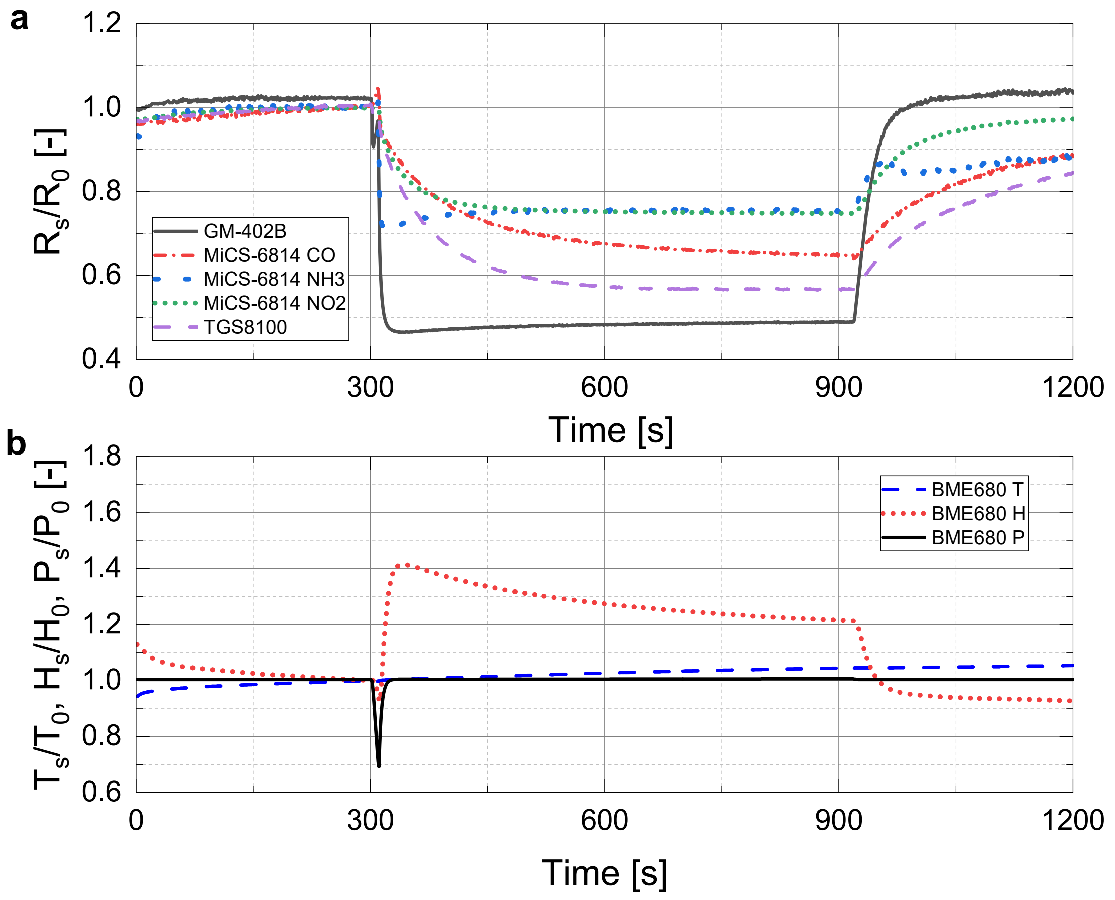

Figure S2. Exemplary time series of (a) DC sensor resistance R_S relative to its initial value R_0 for the applied gas sensor, and (b) environmental conditions (temperature T, humidity H, and pressure P) related to their initial values (T_0, H_0, and P_0 respectively) for a healthy patient. The legend shows the applied sensor according to the names in Table S3 representing the detected gases or environmental conditions.

3. Matlab scripts

The Matlab scripts were used to evaluate four parameters of DC resistance changes: F1, F2, F3, and F4. We applied the following scripts for these parameters:

F1: *delta(1,1)= resToR0(index_end,1)-resToR0(index_begin,1)*;

F2: *center_slope(1,1)= (resToR0(index_end,1)-resToR0(index_end-400,1))/400*; //(determined for 400 samples)

F3: *rising_slope(1,1)= (resToR0(index_end+30,1)-resToR0(index_end,1))/30*; //(30 samples since *index_end*)

F4: *falling_slope(1,1)=(resToR0(index_begin+30,1)-resToR0(index_begin,1))/30*; //(30 samples since *index_begin*)

where *resToR0* is a table of recorded relative resistances R_S/R_0; *index_end* is an index (number of the recorded sample) responding to the end of the analysing phase; *index_begin* is an index (number of the recorded sample) responding to the beginning of the analysing phase; both indexes (*index_end* and *index_begin*) were recorded automatically at the moments of switching the pump and valves, controlled by electronic circuit and software. The detailed data of the evaluated F1, F2, F3, and F4 parameters for all applied sensors are available under email request from the corresponding author: janusz.smulko@pg.edu.pl. The estimated values of the parameters F1, F2, and F3 for the sensors TGS8100 and MiCS-6814 (CO) are presented in Figure S3 and Figure S4 respectively.

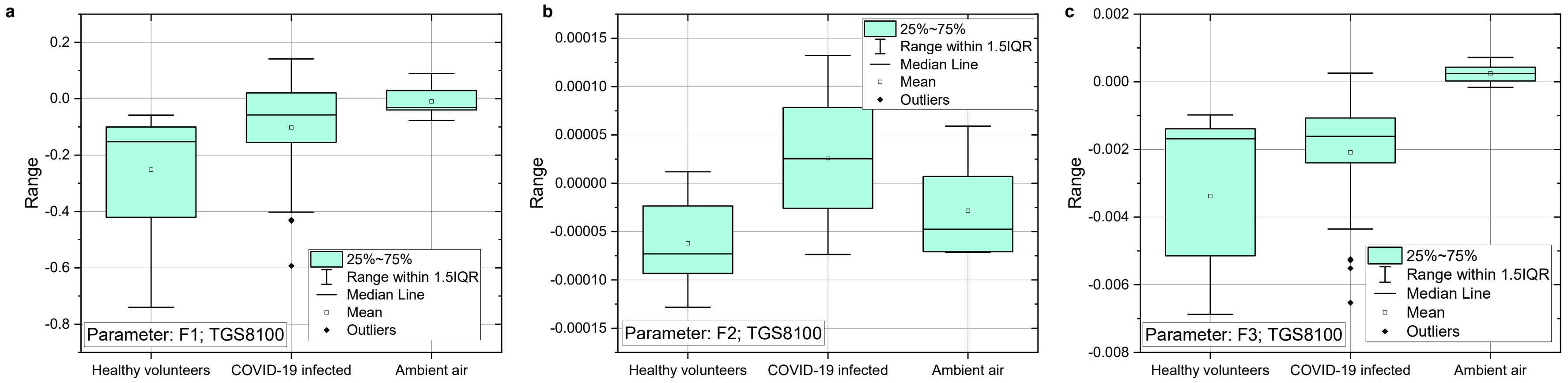

Figure S3. Difference in response of the TGS8100 gas sensor between the groups of COVID-19-infected patients (33), healthy volunteers (17) and ambient air (6) for three selected parameters of major difference: (a) F1, (b) F2, and (c) F3. IQR –interquartile range, presenting the statistical dispersion of the considered data.

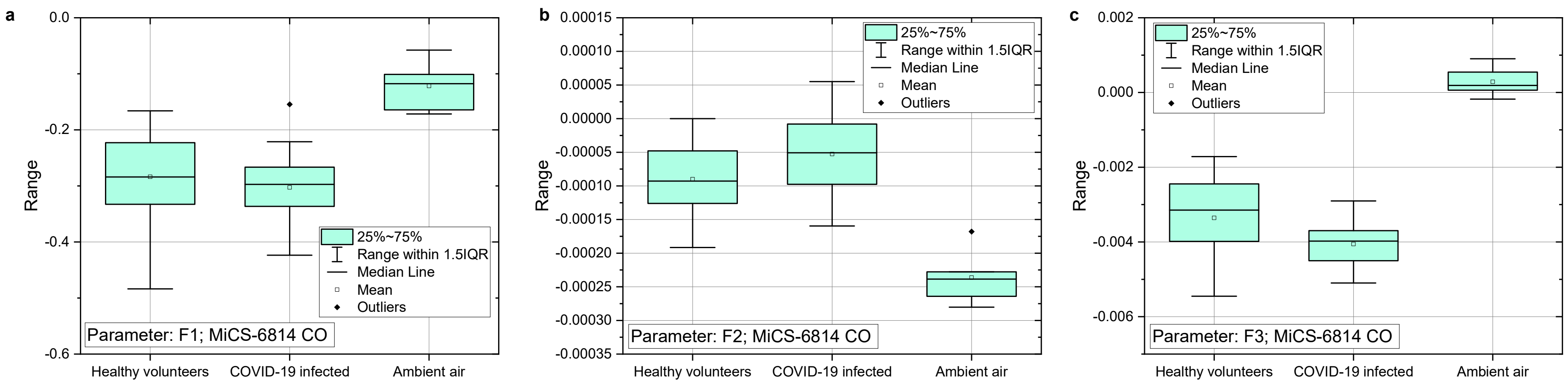

Figure S4. Difference in response of the MiCS-6814 (CO) gas sensor between the groups of COVID-19-infected patients (33), healthy volunteers (17) and ambient air (6) for three selected parameters of major difference: (a) F1, (b) F2, and (c) F3. IQR –interquartile range, presenting the statistical dispersion of the considered data.

4. Detection efficiency

We present here the detailed tables of detection efficiency by the considered algorithms. The tables were derived by the *Test and Score* widget in the Orange software. The presented parameters mean:

- **The area under ROC** – the area under the ROC curve.
- **Classification accuracy** – the proportion of correctly classified data.
- **Precision** – the proportion of true positives among cases classified as positive.
- **Recall** – the proportion of true positives among all positive instances in the data.
- **Specificity** – the number of non-sick among all diagnosed as non-sick.
- **LogLoss** – cross-entropy loss (the uncertainty of the received prediction based on how much it varies from the actual label).

Table S4. The detailed detection results were obtained for the examined cohort of 50 samples (33 COVID-19-infected patients, 17 healthy volunteers) by the four considered algorithms: SVM, kNN, Random Forest, Neural Network. The presented results consider the data used for the ROC curve presented in Figure 5.

Algorithm	Area under ROC	Classification accuracy	Precision	Recall	LogLoss	Specificity
SVM	0.872	0.820	0.818	0.820	0.416	0.765
Neural Network	0.848	0.840	0.838	0.840	0.564	0.775
Random Forest	0.830	0.840	0.840	0.840	1.703	0.803
kNN	0.830	0.700	0.696	0.700	0.440	0.617

Table S5. The detailed detection results were obtained for the examined cohort of 24 samples (16 COVID-19-infected patients over 55 years old, 8 healthy volunteers over 45 years old) by the four considered algorithms: SVM, kNN, Random Forest, Neural Network. The presented results consider the data used for the ROC curve presented in Figure 6a.

Algorithm	Area under ROC	Classification accuracy	Precision	Recall	LogLoss	Specificity
SVM	1.000	0.958	0.961	0.958	0.141	0.917
Neural Network	1.000	0.958	0.963	0.958	0.101	0.979
kNN	0.930	0.917	0.917	0.917	0.326	0.896
Random Forest	0.930	0.917	0.926	0.917	0.295	0.833

Table S6. The detailed detection results were obtained for the examined cohort of 26 samples (17 COVID-19-infected patients under 55 years old, 9 healthy volunteers under 45 years old) by the four considered algorithms: SVM, kNN, Random Forest, Neural Network. The presented results consider the data used for the ROC curve presented in Figure 6b.

Algorithm	Area under ROC	Classification accuracy	Precision	Recall	LogLoss	Specificity
kNN	0.886	0.846	0.847	0.846	0.427	0.762
Random Forest	0.886	0.846	0.846	0.846	0.389	0.814
SVM	0.863	0.808	0.812	0.808	0.551	0.689
Neural Network	0.908	0.808	0.804	0.808	0.458	0.741
